# Preliminary feasibility of pre-treatment F-18-PSMA-1007 PET/CT in dose prediction for Lu-177-PSMA-I&T therapy

**DOI:** 10.2340/1651-226X.2026.45700

**Published:** 2026-07-22

**Authors:** Suvi Kokkonen, Vappu Reijonen, Eero Hippeläinen, Veera Ahtiainen, Jukka Schildt, Outi Sipilä, Sauli Savolainen, Mikko Tenhunen

**Affiliations:** a Cancer Center, Helsinki University Hospital, Helsinki, Finland; b Department of Physics, University of Helsinki, Helsinki, Finland; c Clinical Physiology and Nuclear Medicine, Helsinki University Hospital, Helsinki, Finland

**Keywords:** Castration-resistant prostate cancer, PSMA-PET, [177Lu]Lu-PSMA radioligand therapy, theranostic

## Introduction

In metastatic castration-resistant prostate cancer (mCRPC), the treatment options are limited to palliative approaches, including targeted radionuclide therapy (TRT) using Lutetium-177 prostate-specific membrane antigen ([177Lu]Lu-PSMA) [1, 2]. Eligibility for [^177^Lu]Lu-PSMA therapy is commonly assessed with a pre-treatment Fluorine-18 [^18^F]F-PSMA positron emission tomography/computed tomography (PET/CT) to confirm sufficient PSMA accumulation in tumor lesions [3].

TRT is typically administered with fixed activity, not accounting for interpatient variability in biodistribution and biokinetics, leading to potential under- or overtreatment in some patients [4, 5]. The kidneys are considered critical organs in [^177^Lu]Lu-PSMA therapies, in addition to red bone marrow, salivary, and lacrimal glands. Patient-specific dosimetry is recommended [6].

There is significant variation in the reported dose estimates, which may be not only related to patient characteristics but also to the influence of heterogenous methodological factors [7]. Post-treatment dosimetry in [^177^Lu]Lu-PSMA therapy is typically performed using single-photon emission computed tomography/computed tomography (SPECT/CT), but the imaging time points, acquisition and reconstruction protocols, and segmentation methods vary between centers. There are initiatives for harmonization [8–10]. Several studies have addressed the predictive power of pre-therapy PSMA PET imaging with promising, yet varying results [11–13]. No established method exists to use pre-therapy PET imaging for individualized dose planning in PSMA TRT.

Recent studies report correlations between the [^177^Lu]Lu-PSMA absorbed dose to tumor, the pre-treatment PSMA PET/CT uptakes, and treatment response, suggesting the treatment response could be refined with a dosimetric approach [14, 15]. Preliminarily, we hypothesize that pre-treatment PSMA PET/CT may provide quantitative information to predict absorbed doses. Exploration of the technical factors is a mandatory step preceding this, and a pilot approach was taken.

## Patients/material and methods

### Patient data

This retrospective feasibility study included five mCRPC patients who underwent both [^18^F]F-PSMA ([^18^F]F-PSMA-1007) pre-treatment PET/CT imaging and [^177^Lu]Lu-PSMA ([^177^Lu]Lu-PSMA-I&T) therapy followed by two time-point post-treatment SPECT/CT imaging at the Hospital District of Helsinki (HUS).

[^177^Lu]Lu-PSMA therapy was carried out with an administered activity of 7.4 GBq (± 5%) per cycle. Dosimetry results from the first therapy cycle are studied.

In total, 10 kidney and six bone lesion volumes of interest (VOI) were analyzed. The patient-specific administered activities and CT-based volumes of kidneys and bone lesions are summarized in Table 1. The methodological workflow is presented in Supplementary Appendix A.

**Table 1:** Administered activities of [^18^F]F-PSMA for pre-treatment PET/CT and [^177^Lu]Lu-PSMA treatment, including the CT-based segmented volumes for kidneys, denoted as K_L_ for left and K_R_ for right, and bone lesions, denoted as B_L_.

	[^18^F]F-PSMA	PET/CT V_CT_ [mL]			[^177^Lu]Lu-PSMA	SPECT/CT V_CT_ [mL]		

Patient #	A [MBq]	K_L_	K_R_	BL	A [GBq]	K_L_	K_R_	BL
1	219	147	117	3.8	7.5	163	160	3.7
2	301	249	236	11.2	7.6	258	240	10.4
3 (1)	219	141	131	16.4	7.3	150	164	6.3
3 (2)				9.6				6.6
4	213	121	145	18.0	7.1	132	145	20.8
5	244	116	147	0.5	7.4	119	168	0.9
Median (range)	219 (213–301)	143 (116–249)		10.4 (0.5–18.0)	7.4 (7.1–7.6)	165.5 (119–258)		6.5 (0.9–20.8)

PSMA: Prostate-specific membrane antigen; PET/CT: Positron emission tomography/computed tomography; V_CT_: CT-based delineated volume; SPECT: Single-photon emission computed tomography; K_L_: Left kidney; K_R_: Right kidney; B_L_: Bone lesion.

### Imaging and reconstruction protocols

PET/CT imaging was performed approximately 90 min post [^18^F]F-PSMA injection. Two SPECT/CT scans were taken approximately 24 h and a week post-treatment [6].

For PET/CT, either GE Healthcare Discovery MI (GE Healthcare, Chicago, IL, USA) or Siemens Biograph mCT 64 R4 (Siemens Healthcare, Erlangen, Germany) was used with EARL2-compliant reconstructions [16]. For SPECT/CT, Siemens SYMBIA pro.Specta Q3 (Siemens Healthcare, Erlangen, Germany) was used with Ordered Subsets Conjugate Gradient Minimizer (OSCGM) reconstruction [17]. Imaging and reconstruction parameters are listed in Appendix B.

### Segmentation

The kidneys and bone lesions were segmented separately on the PET/CT and SPECT/CT images, using CT- and gradient-based approaches. CT-based kidney segmentation was performed using MIM Software’s ProtegeAI+ workflow, while lesions were manually segmented on CT [18]. Gradient-based segmentation for both kidneys and lesions was performed using the PET/SPECT intensity gradient-based PET Edge tool in MIM Software [19]. Only lesions clearly visible in PET, SPECT, and CT images were included.

### Recovery coefficients

Recovery coefficient (RC) correction was applied to activity uptakes on CT-based bone lesion volumes to improve post-therapy SPECT scan’s quantitative accuracy. RCs for SPECT’s OSCGM reconstruction were determined using the standard International Electrotechnical Commission (IEC) Body Phantom from the National Electrical Manufacturers Association (NEMA) [20]. The phantom spherical inserts were filled with ^177^Lu water solution (6.6 ± 0.2 MBq/g), followed by SPECT/CT scan using Siemens Pro.Specta Q3 [21]. Detailed imaging parameters are found in Supplementary Appendix B.

### Dosimetry and dose prediction

Post-therapy absorbed doses were determined based on two-time-point SPECT/CT imaging and implemented in MIM Software [5].

Pre-treatment dose predictions were derived from PET/CT using an experimental dosimetry module (Madsen Dosimetry Estimator, MIM Software) [22], which extrapolates absorbed dose from pre-therapy activity distributions under specified effective half-life assumptions, detailed below.

Three different effective half-lives for kidneys and bone lesions were tested: patient-specific values observed from post-therapy SPECT/CT dosimetry (CT-based volumes and OSCGM reconstruction), and two literature-based values by Schuchardt et al. (33 h for kidneys and 43 h for bone lesions) [23], and Karimzadeh et al. (39 h for kidneys and 56 h for bone lesions) [24]. The patient-specific post-therapy dosimetry results and effective half-lives were considered as the reference, while literature half-life-based predictions were evaluated against these to assess the feasibility of dose prediction without the prior knowledge of individual kinetics.

## Results

### Recovery coefficients

SPECT recovery coefficients were modeled using monoexponential fits. For OSCGM reconstruction, the recovery curve was:

*RC*=1.090-1.607*e*^-0.052^^*D*^

where *D* is the sphere diameter (mm). The RC measurements and the model fit are presented in Supplementary Appendix C.

### Post-therapy dosimetry using SPECT/CT

The kidneys’ median absorbed dose using OSCGM reconstruction and CT-based segmentation was 3.1 Gy (range 2.0–5.9 Gy). For bone lesions, the median absorbed dose was 33.8 Gy (range 10.4–68.9 Gy). Patient-specific results can be found in Table 2 (under D_ref_).

**Table 2:** Absorbed dose predictions (D_Schuchardt_) derived from pre-treatment PET/CT scan, using CT-based segmentation and literature effective half-lives [23] for kidneys (t_Schuchardt_ = 33 h) and bone lesions (t_Schuchardt_ = 43 h).

Kidneys								
	PET/CT		SPECT/CT				Ratio of doses from PET/SPECT	
	D_Schuchardt_ [Gy]		D_ref_ [Gy]		t_ref_ [h]		D_Schuchardt_/D_ref_	
Patient	L	R	L	R	L	R	L	R
1	7.5	6.6	3.1	3.1	30	32	2.4	2.1
2	5.0	5.8	2.0	2.4	28	29	2.5	2.4
3	4.5	2.8	3.7	4.3	35	30	1.2	0.7
4	5.5	5.5	2.0	2.1	32	30	2.8	2.6
5	4.6	6.0	4.1	5.9	29	29	1.1	1.0
Median (range)	5.5 (2.8–7.5)		3.1 (2.0–5.9)		30 (29–35)		2.3 (0.7–2.8)	
Bone lesions								
1	1.5		10.4		67		0.1	
2	13.7		21.2		61		0.6	
3 (1)	28.1		68.9		159		0.4	
3 (2)	14.2		49.2		159		0.3	
4	24.6		46.3		61		0.5	
5	23.9		16.7		32		1.4	
Median (range)	19.1 (1.5–28.1)		33.8 (10.4–68.9)		64 (32–159)		0.5 (0.1–1.4)	

PET: Positron emission tomography; SPECT: Single-photon emission tomography.

SPECT/CT-based mean absorbed doses (D_ref_) and effective half-lives (tref) are reported using CT-based segmentation and OSCGM reconstruction. Activity uptakes on CT-based lesion volumes have been RC corrected for post-therapy dosimetry results. Prediction performance is expressed as the ratio of PET/CT-based predicted doses to SPECT/CT-based observed doses.

Using OSCGM reconstruction and gradient-based segmentation, the kidney median absorbed dose was 2.7 Gy (range 1.8–8.9 Gy), and the median bone lesion absorbed dose was 20.6 Gy (6.5–44.5 Gy).

### Pre-therapy dose estimation using PET/CT

Table 2 summarizes the key findings: PET/CT-based absorbed dose predictions for kidneys and bone lesions using literature effective half-lives [23], with SPECT/CT-based observed absorbed doses and corresponding effective half-lives, using current local clinical methodology. Here, the literature half-lives [23] were chosen as the study included more patients (51 patients in [23] vs. 16 in [24]). The median ratio between PET-predicted and SPECT-observed absorbed doses for kidneys was 2.3 (range 0.7–2.8). For lesions, the corresponding median ratio was 0.5 (range 0.1–1.4).

Using the literature effective half-lives [24], the median ratio for kidneys was 2.8 (range 0.8–3.6) and for lesions 0.6 (range 0.2–1.8). Using the reference effective half-lives in PET prediction, the median ratio was 2.3 (range 0.6–3.2) for kidneys and 1.0 (range 0.2–1.5) for lesions.

Dose predictions for combinations of the three effective half-lives and the two segmentation methods are presented in detail in Supplementary Appendix D and E.

## Discussion

This pilot study evaluated the feasibility of pre-treatment dosimetry from single time-point [^18^F]F-PSMA-1007 PET/CT to predict absorbed doses in [^177^Lu]Lu-PSMA-I&T therapy. The main preliminary finding is that PET-based dose prediction tended to overestimate kidney absorbed dose and underestimate the bone lesion absorbed dose, with substantial interpatient and inter-lesion variability. The segmentation methods produced more consistent dosimetry results for kidneys, while greater variability was observed in lesions. The median absorbed doses were comparable to those reported in previous studies [14, 23–28].

Clearly, our pilot approach had limitations. First, the study had a limited patient cohort, and consequently such a limited sample size has no statistical power. The number of analyzed lesions per patient was limited due to the selection criteria, leading to potential high uncertainty. Further studies with a larger sample size are needed.

Furthermore, only the low-dose, noncontrast CT scans were utilized with PET and SPECT imaging. Using diagnostic CT and MRI scans could improve the accuracy of lesion delineation. The volumes of the lesions analyzed were below 21 mL, and the partial volume effect limits accurate activity estimation. Basic RC correction was carried out for post-therapy SPECT/CT scans with diligent registration to counterbalance these effects. PET-based lesion dose predictions could be improved by applying similar corrections. Despite EARL2 reconstructions, the use of two different PET/CT systems may affect the quantitation.

The SPECT-observed effective half-lives for the kidneys aligned more closely with the first set of literature-reported effective half-lives [23] than with the second set [24]. On the other hand, the SPECT-observed mean effective half-lives for bone lesions resembled the second set of literature values [24] more than the first [23]. Especially the SPECT-observed bone lesion effective half-lives exhibited a wide range of values, speaking for the need of using patient- or tumor-specific effective half-lives to achieve better accuracy in dose prediction. The accuracy of post-treatment dosimetry results could be improved using SPECT/CT scans from more time points. The results could also improve, for instance, with population pharmacokinetic modeling [29].

An avenue to explore could be to find systematic correction factors that can be applied to reduce the bias between predicted and calculated absorbed doses. This concept was researched by Peters et al. [30] using pre-treatment ^68^Ga-PSMA-11 PET imaging to predict the absorbed dose in [^177^Lu]Lu-PSMA-617 treatment. They reported a scaling factor of 2.2 for kidneys. Lesions showed large interpatient variation. We could not determine such tentative PET/SPECT correction factors in this pilot study.

The predictive power of pre-treatment PET imaging has been studied more extensively in the case of somatostatin receptor radionuclide therapy with statistically significant yet modest results [31]. The kinetic difference between the short-lived PET tracers and the therapy agents ([^18^F]F-PSMA-1007 vs. [^177^Lu]Lu-PSMA-I&T [32]) may present a limitation that cannot be fully resolved. The short physical half-life of ^18^F limits the time-point of PET/CT imaging, giving an image of a relatively early distribution and clearance. A potential solution to address this issue could be using a longer-lived PET or SPECT-compatible isotope to enable imaging at later time-points [33, 34].

## Conclusion

In this feasibility study, dose prediction for the first [^177^Lu]Lu-PSMA-I&T treatment cycle based on [^18^F]F-PSMA-1007 PET/CT overestimated kidney absorbed dose and underestimated bone lesion absorbed dose compared to post-treatment SPECT/CT-based absorbed doses. Observed substantial interpatient variation suggests caution in PET-based dose prediction and the need for further studies.

## Supplementary Material



## Data Availability

Research data are stored in an institutional repository and will be shared upon reasonable request to the corresponding author.
