# Supplementary material for: Preliminary feasibility of pre-treatment F-18-PSMA-1007 PET/CT in dose prediction for Lu-177-PSMA-I&T therapy
Source: Acta Oncol. 2026 Jul 22;65:45700. doi: 10.2340/1651-226X.2026.45700 (PMC13402945; doi:10.2340/1651-226X.2026.45700)
Supplement: Supplementary file 1 [file AO-65-45700-s1.pdf]

**Supplementary material has been published as submitted. It has not been copyedited, or typeset by Acta Oncologica**

Supplementary Material

Appendix A:

Figure A: The workflow of methods used.

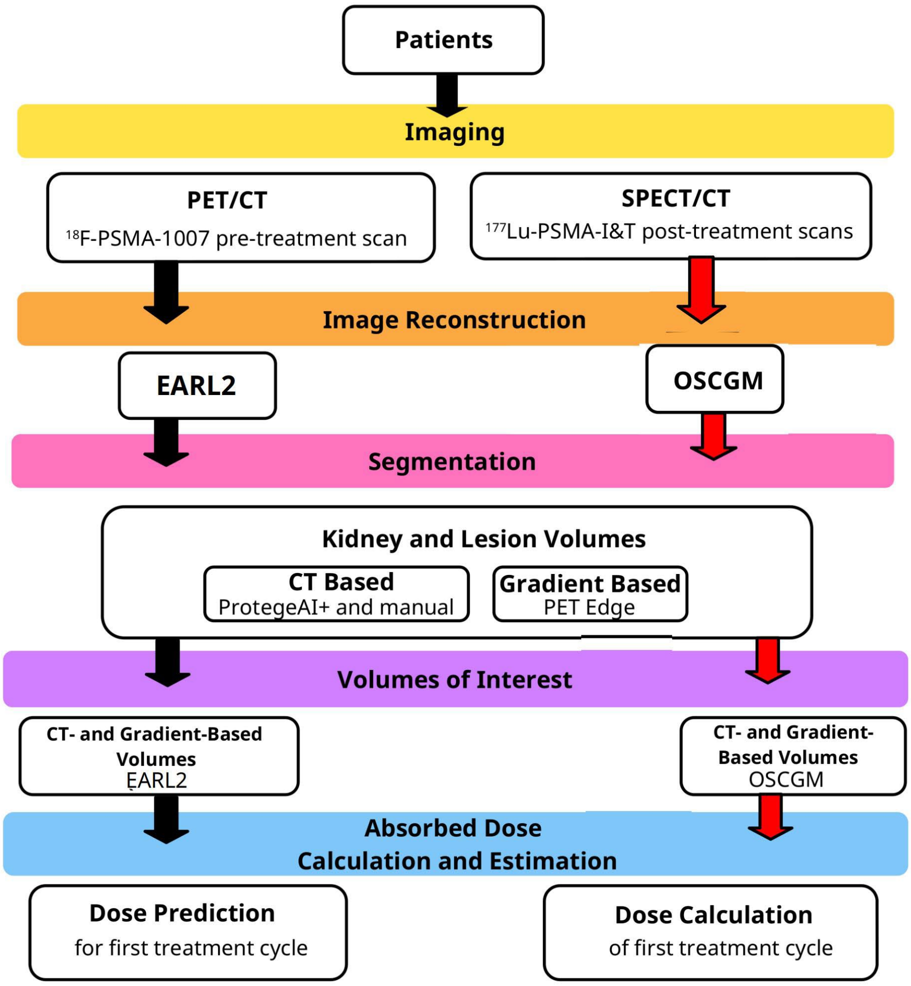

Appendix B:

Table B: Imaging/reconstruction parameters. The patients' pre-treatment scanning was carried out with two different PET/CTs (EARL2 accredited), and post-treatment scanning with one SPECT/CT.

| Parameter | GE Healthcare Discovery MI PET/CT | Siemens Biograph mCT 64 R4 PET/CT | Siemens SYMBIA pro.Specta Q3 SPECT/CT with 5/8" crystals and MELP collimators |
|---|---|---|---|
| Axial field of view | 20 cm | 21.6 cm | 38.7 cm |
| Acquisition time | 2:45 min / bed position | flow table: 1 mm/s | 60 views, 10 s per view |
| Scan range | Orbits to thighs | Orbits to thighs | 3 bed positions from the crown of the head |
| Reconstruction | OSEM + TOF | OSEM + TOF | OSCGM |
| Iterations/Subsets | 3/16 | 2/21 | 8/4 |
| Post-filtering | Gaussian filter 6.0 mm | Gaussian filter 5.5 mm | Gaussian filter 8.4 mm |
| Corrections | CT based AC, scatter, PSF, random, dead time | CT based AC, scatter, PSF, random, dead time | CT based AC, scatter, dead time, Siemens BroadQuantTM |
| Matrix size | 256 × 256 | 400 x 400 | 256 × 256 |
| Slice Thickness | 5 mm | 5 mm | 3 mm |

Appendix C:

Table C: The phantom's insert diameter and the calculated recovery coefficient, using OSCGM reconstruction and CT-based segmentation based on the known diameters. The phantom was SPECT/CT imaged using Siemens Pro.Specta Q3.

| Diameter [mm] | Volume [ml] | RC |
|---|---|---|
| 10 | 0.5 | 0.15 |
| 13 | 1.2 | 0.26 |
| 17 | 2.6 | 0.43 |
| 22 | 5.6 | 0.56 |
| 28 | 11.5 | 0.75 |
| 37 | 26.5 | 0.84 |

Figure C: The recovery coefficients were fitted using a monoexponential model, shown in the Figure. The fitted model describes the data well with $R^2 = 0.995$.

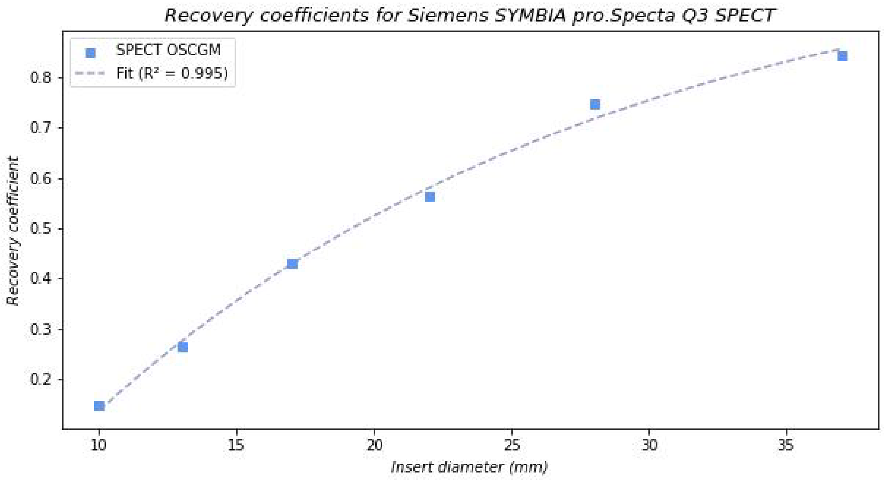


Appendix D:

Table D: Absorbed dose predictions derived from pre-treatment PET/CT scan using SPECT observed effective half-lives ($D_{SPECT/CT}$), literature values reported by Schuchardt et al. [23] ($D_{Schuchardt}$), and literature values reported by Karimzadeh et al. [24] ($D_{Karimzadeh}$) for CT-based and gradient-based kidney and bone lesion volumes. Kidneys are denoted as L for left kidney and R for right kidney. SPECT/CT observed mean absorbed doses ($D_{ref}$) for both segmentation methods are presented as well.

| PET/CT | | | | | | | SPECT/CT | |
|---|---|---|---|---|---|---|---|---|
| CT-Based Segmentation | | | | | | | CT-Based Segmentation | |
| Kidneys | | | | | | | Kidneys | |
| | $D_{SPECT/CT}$ [Gy] | | $D_{Schuchardt}$ [Gy] | | $D_{Karimzadeh}$ [Gy] | | $D_{ref}$ [Gy] | |
| Patient | L | R | L | R | L | R | L | R |
| 1 | 7.4 | 6.9 | 7.5 | 6.6 | 8.8 | 8.8 | 3.1 | 3.1 |
| 2 | 4.6 | 5.4 | 5.0 | 5.8 | 5.8 | 6.8 | 2.0 | 2.4 |
| 3 | 5.0 | 2.6 | 4.5 | 2.8 | 5.3 | 3.3 | 3.7 | 4.3 |
| 4 | 6.3 | 5.4 | 5.5 | 5.5 | 7.1 | 6.5 | 2.0 | 2.1 |
| 5 | 4.2 | 5.5 | 4.6 | 6.0 | 5.4 | 7.1 | 4.1 | 5.9 |
| Bone lesions | | | | | | | | |

| | | | | | | | | |
|---|---|---|---|---|---|---|---|---|
| 1 | 2.3 | | 1.5 | | 1.9 | | 10.4 | |
| 2 | 19.2 | | 13.7 | | 17.4 | | 21.2 | |
| 3 (1) | 102.1 | | 28.1 | | 35.7 | | 68.9 | |
| 3(2) | 51.5 | | 14.2 | | 18.0 | | 49.2 | |
| 4 | 34.7 | | 24.6 | | 31.3 | | 46.3 | |
| 5 | 18.2 | | 23.9 | | 30.4 | | 16.7 | |
| Gradient-Based Segmentation | | | | | | | | |
| Kidneys | | | | | | | | |
| | $D_{SPECT/CT}$ [Gy] | | $D_{Schuchardt}$ [Gy] | | $D_{Karimzadeh}$ [Gy] | | $D_{ref}$ [Gy] | |
| | L | R | L | R | L | R | L | R |
| 1 | 6.6 | 7.2 | 6.6 | 6.9 | 7.8 | 7.8 | 2.8 | 2.6 |
| 2 | 4.3 | 4.8 | 4.7 | 5.1 | 5.5 | 6.0 | 1.8 | 2.1 |
| 3 | 4.5 | 2.6 | 4.1 | 2.8 | 4.8 | 3.3 | 3.2 | 5.5 |
| 4 | 5.6 | 5.1 | 5.5 | 5.3 | 6.4 | 6.3 | 1.9 | 2.3 |
| 5 | 3.3 | 4.5 | 4.1 | 5.6 | 4.8 | 6.6 | 6.6 | 8.9 |
| Bone Lesions | | | | | | | | |
| 1 | 5.1 | | 3.7 | | 4.7 | | 6.5 | |
| 2 | 19.2 | | 11.6 | | 14.7 | | 14.5 | |
| 3 (1) | 103.4 | | 28.4 | | 36.2 | | 39.6 | |
| 3 (2) | 49.5 | | 13.6 | | 17.3 | | 26.7 | |
| 4 | 33.3 | | 24.0 | | 30.6 | | 44.5 | |
| 5 | 21.4 | | 28.9 | | 36.7 | | 3.3 | |

Appendix E: Ratio between the PET/CT-based absorbed dose predictions ($D_{SPECT/CT}$, $D_{schuchardt}$, $D_{karimzadeh}$ and the reference SPECT/CT-based observed absorbed dose ($D_{ref}$) for kidneys, denoted as L and R for left and right kidney, and bone lesions for the two segmentation methods used (CT- and gradient-based segmentation).

| Ratio of absorbed doses from PET/SPECT | | | | | | |
|---|---|---|---|---|---|---|
| CT-Based Segmentation | | | | | | |
| Kidneys | | | | | | |
| | $D_{SPECT/CT}/D_{ref}$ | | $D_{Schuchardt}/D_{ref}$ | | $D_{Karimzadeh}/D_{ref}$ | |
| Patients | L | R | L | R | L | R |
| 1 | 2.4 | 2.2 | 2.4 | 2.1 | 2.8 | 2.8 |
| 2 | 2.3 | 2.3 | 2.5 | 2.4 | 2.9 | 2.8 |
| 3 | 1.4 | 0.6 | 1.2 | 0.7 | 1.4 | 0.8 |
| 4 | 3.2 | 2.6 | 2.8 | 2.6 | 3.6 | 3.1 |
| 5 | 1.0 | 0.9 | 1.1 | 1.0 | 1.3 | 1.2 |

| | | | | | | |
|---|---|---|---|---|---|---|
| Median (range) | 2.3 (0.6 – 3.2) | | 2.3 (0.7 – 2.8) | | 2.8 (0.8 – 3.6) | |
| Bone Lesions | | | | | | |
| 1 | 0.2 | | 0.1 | | 0.2 | |
| 2 | 0.9 | | 0.6 | | 0.8 | |
| 3 (1) | 1.5 | | 0.4 | | 0.5 | |
| 3 (2) | 1 | | 0.3 | | 0.4 | |
| 4 | 0.7 | | 0.5 | | 0.7 | |
| 5 | 1.1 | | 1.4 | | 1.8 | |
| Median (range) | 1.0 (0.2 – 1.5) | | 0.5 (0.1 – 1.4) | | 0.6 (0.2 – 1.8) | |
| Gradient-Based Segmentation | | | | | | |
| Kidneys | | | | | | |
| | $D_{SPECT/CT}/D_{ref}$ | | $D_{Schuchardt}/D_{ref}$ | | $D_{Karimzadeh}/D_{ref}$ | |
| | L | R | L | R | L | R |
| 1 | 2.3 | 2.8 | 2.4 | 2.7 | 2.8 | 3.0 |
| 2 | 2.4 | 2.2 | 2.6 | 2.4 | 3.1 | 2.8 |
| 3 | 1.4 | 0.5 | 1.3 | 0.5 | 1.5 | 0.6 |
| 4 | 2.9 | 2.2 | 2.8 | 2.3 | 3.3 | 2.7 |
| 5 | 0.5 | 0.5 | 0.6 | 0.6 | 0.7 | 0.7 |
| Median (Range) | 2.2 (0.5 – 2.9) | | 2.4 (0.5 – 2.8) | | 2.8 (0.6 – 3.3) | |
| Bone Lesions | | | | | | |
| 1 | 0.8 | | 0.6 | | 0.7 | |
| 2 | 1.3 | | 0.8 | | 1.0 | |
| 3 (1) | 2.6 | | 0.7 | | 0.9 | |
| 3 (2) | 1.9 | | 0.5 | | 0.6 | |
| 4 | 0.7 | | 0.5 | | 0.7 | |
| 5 | 6.6 | | 8.8 | | 11.2 | |
| Median (Range) | 1.6 (0.7 – 6.6) | | 0.7 (0.5 – 8.8) | | 0.8 (0.6 – 11.2) | |
